# Prison Administration and the Training of Officials.—IV

**Published:** 1898-04-16

**Authors:** 


### Modern Sociology.

### PRISON ADMINISTRATION AND THE TRAINING OF OFFICIALS.—IV.

In dealing hitherto with the subject of prison administration and the training of officials we have been describing the measures which have already been taken in that direction by the authorities at the Home Office, and which, although as yet only carried out to a very inadequate extent, are still admirable in their commencement and full of promise for the future. Now, however, we have to turn to a form of training and preparation for prison duties which has never been dreamt of as desirable, much less as a necessity, inasmuch as it applies to those high-class functionaries to whom the moral and spiritual welfare of the criminals is committed. Nevertheless, we speak from the experience of many years' attendance at the religious services provided for prisoners, and we may therefore claim some right to judge critically of the discourses habitually delivered before them, and on which they have often commented to ourselves in very significant terms. We are well assured that there does not exist a more estimable or devoted body of men than the clergymen who are appointed to the chaplaincies of our prisons all over the country; they are, as we have known them, most assiduous in their duties as regards visiting the prisoners in their cells. What species of instruction may be given to the criminals in these private interviews is known only to the visitor and the visited, and we must hope that it may always tend to edification, but it is of the sermons delivered to the prisoners *en masse* in the Sunday services that we have now to speak. We have had opportunities of hearing some hundreds of these discourses, and we do not hesitate to say that, with very rare exceptions, they have been singularly unsuited to the audience, and of a nature likely to prove completely barren of good results. The hearers whom these gentlemen are asked to instruct consist of a *mélange* of professional thieves, habitual drunkards, murderers awaiting their execution, and vagabonds of every description, illiterate, lawless, and for the most part absolutely without religious knowledge or belief. They are assembled there, compelled to listen to the words of the servants of God, which outside the prison walls they would certainly never seek to hear—and a most splendid opportunity it is to teach them in plain emphatic language what is presumably essential to their well-being, the very foundations of religious truth, of personal righteousness, of their own responsibilities as sentient beings who will one day have to face death, and give an account hereafter of the lives they are now squandering in guilt and corruption. Instead of such teaching as this, the good chaplains seem to assume that their convict hearers are as well read in Scripture and in all the tenets of the Christian faith, as an ordinary well-educated and respectable congregation in a city church might be, and they fly over their heads with dissertations in language more or less incomprehensible to them, on some passages in the Bible which could only be understood by a clear previous knowledge of the subject. Almost invariably the preacher selects some character or incident from the lesson for the day, and discourses upon it in a descriptive and discursive manner as if the context was duly known and appreciated by the audience, to whom—as a matter of fact—the subject conveys no meaning of any kind whatever. A chaplain in our hearing once proceeded thus, after a few remarks on a portion of the Old Testament: "Let us now consider, my brethren, what were the characteristics of Ezra." It is doubtful whether many of the hearers realised that he was even talking of a man and not of some unknown animal of which they had never heard. On another occasion, after intricate and involved comments on an abstruse verse from one of the epistles, the prisoners were invited to form a clear conception "of the mind of Paul." We need not multiply instances of this kind. As a rule, these sermons are generally much too discursive. Instead of taking one fundamental truth, and explaining it and it alone in the simplest possible language, the preachers are apt to wander off into difficult themes which have very little connection with their text, so that in the end their hearers have not the remotest idea what they have been talking about. In recommending that chaplains should be trained for their very peculiar functions, it must be remembered that they are often very young men, as it is considered quite allowable when the vicar of a parish is appointed to any provincial prison as chaplain that he should send his curates to take the prison services while he is employed in his own church. If it is asked by what means prison chaplains can be trained, the answer is very simple. There are experienced and hard-working clergymen in the East-end of London and in all our large towns who encounter in the slums they have to deal with just such lawless, ignorant, and corrupt individuals as furnish the material for our prison populations. Let the future chaplain go to work for some months in a district peopled by those who have been harshly termed the "scum of the earth" under the wise direction of one who has long been accustomed to labour among them, and he will soon learn what species of instruction is required for the prisoners who will be committed to his care. We urge this most earnestly, because a really clear and judicious sermon such as is sometimes preached by wise men in prisons is extremely valuable. We gladly record a striking instance of this fact. An elderly man in prison, who had passed many years of his life between receptacles of that kind and workhouses, had sunk into the lowest depths of hopelessness to such an extent that he made a desperate effort to starve himself to death, which the prison officials had much trouble in frustrating. The man was in chapel when a preacher of much experience, acting temporarily for the chaplain, took for his theme the statement that God helped those who helped themselves, and gave a most clear exposition of it suited to the capacities of those around him. The broken-down man listened with rapt attention, and when the governor visited him afterwards in his cell he exclaimed, "Sir, that parson has come here for me." The preacher, hearing of this remark, went to see and advise him, and the result was that the prisoner regained hope and courage. He became, as he said himself, "a new man," prepared to do honest work on his release, and fight the battle of life with heart and energy.

